# Intake of *Lactiplantibacillus plantarum* HEAL9 Improves Cognition in Moderately Stressed Subjects: A Randomized Controlled Study

**DOI:** 10.3390/nu15153466

**Published:** 2023-08-05

**Authors:** Gunilla Önning, Caroline Montelius, Magnus Hillman, Niklas Larsson

**Affiliations:** 1 Biomedical Nutrition, Pure and Applied Biochemistry, Lund University, 222 00 Lund, Sweden; 2 Probi AB, 223 70 Lund, Sweden; caroline.montelius@probi.com (C.M.);; 3 Diabetes Research Laboratory, Department of Clinical Sciences Lund, Lund University, 221 84 Lund, Sweden; magnus.hillman@med.lu.se

**Keywords:** probiotics, *L. plantarum*, stress, gut–brain axis, working memory, cognition, mood, sleep, tryptophan, inflammation

## Abstract

Background: The usage of probiotics has expanded beyond the areas of gut and immune health improvement. Several studies have shown the positive impact associated between probiotics and stress, cognition, and mood; a relationship referred to as the gut–brain axis. Method: The aim of this exploratory study was to evaluate the effect of the probiotic strain *Lactiplantibacillus plantarum* HEAL9 (LPHEAL9) on the gut–brain axis in subjects with moderate stress. One hundred and twenty-nine subjects aged 21–52 years completed the study, randomized to consume either LPHEAL9 (*n* = 65) or placebo (*n* = 64) for 12 weeks. Results: Perceived stress and awakening cortisol were significantly reduced over time in both groups. A significant improvement in four cognition tests after consumption of LPHEAL9 compared to placebo was observed (rapid information processing test, numeric working memory test, paired associated learning, and word recall, *p* < 0.05). There was a tendency for a significantly better improvement in the LPHEAL9 group for three mood subscales (Confusion–Bewilderment, Anger–Hostility, and Depression–Dejection) and for fewer subjects with poor sleep in the LPHEAL9 group compared to placebo (*p* < 0.10). Conclusions: Intake of LPHEAL9 significantly improved cognitive functions compared to the placebo, potentially by ameliorating aspects of mood and sleep.

## 1. Introduction

Mental health is one of the fundamental components of health and wellbeing and the COVID-19 pandemic caused an increase in disorders like depression and anxiety [1]. The usage of probiotics may be one way to prevent these problems. Probiotics may affect mental health like cognition and stress via the gut–brain axis. This involves bidirectional communication via neural, endocrine, and immune pathways [2]. Cognitive function encompasses the life-long process of learning and is regulated by the spatial and non-spatial memory, in both rodents and humans [3]. Animal studies have shown that the administration of probiotics to mice and rats is linked to neurogenesis in the hippocampus and an improvement in the associated cognitive functions [3]. The modification of the microbiota by consumption of probiotic bacteria may also affect stress. Intake of probiotics in animal models has been shown to reduce anxiety-like behavior and influence brain activity. In humans, probiotics reduce stress-related gastrointestinal symptoms, circulating proinflammatory cytokines, and the stress hormone cortisol [2]. A placebo-controlled study in healthy but stressed individuals showed that intake of the probiotic strain *Lactiplantibacillus plantarum* HEAL9 (LPHEAL9) for four weeks modified the stress response and reduced novel inflammatory markers during acute stress [4]. The LPHEAL9 strain, combined with S-adenosylmethionine (SAMe), was found to reduce the total score of depression after 2 and 6 weeks of intake in another placebo-controlled study that included subjects with mild-to-moderate depression [5]. The cognitive and anxiety subdomains were also reduced significantly after 2 weeks of consumption. Another *L. plantarum* strain (*Lactiplantibacillus plantarum* 299v, LP299V^®^) has shown positive effects on cognition and mood in subjects with major depression [6]. Intake of LP299V for 8 weeks led to an improvement in the “Attention and Perceptivity Test” and in the “California Verbal Learning Test”, compared with the placebo group (baseline vs. 8 weeks of intervention). The authors linked the effects to a reduction in kynurenine, a compound that is produced during the degradation of tryptophan, which can have a negative effect on cognition. LP299V has also been evaluated in students during examination-induced stress, which showed a positive effect on cortisol levels [7]. 

Two earlier studies in the gut–brain axis area including LPHEAL9 focused on acute stress with measurements on one day [4] or on depressed subjects [5]. To further strengthen the understanding of the gut–brain axis impact of LPHEAL9, an explorative study was performed on healthy subjects with moderate stress who were followed for three months. The aim was to investigate if intake of LPHEAL9 could influence stress, cognition, mood, and quality of sleep. Subjects with moderate stress were included since earlier trials have indicated beneficial effects of probiotics on brain function when the system is challenged with stress [8,9,10]. To evaluate the mechanisms behind the effects, several blood biomarkers were followed, such as the levels of tryptophan, kynurenine, and different markers for inflammation. 

## 2. Materials and Methods

### 2.1. Study Design

The study was a randomized, double-blinded, placebo-controlled, and parallel-designed study, carried out at a single site at Atlantia Clinical Trials, Cork, Ireland, between June 2021 and March 2022. It consisted of a screening visit, followed by a 2 week run-in phase and four assessment visits during a 12 week supplementation phase. Eligible subjects were randomized into the study at visit 2, baseline (week 0). Study assessments carried out during the visits included saliva awakening cortisol, analysis of biomarkers in blood samples, cognitive assessment, perceived stress, quality of sleep, and mood assessment. Subjects also answered questions regarding their lifestyle such as diet, exercise, and job on all occasions to determine if there had been some changes during the study time. Apart from the cognition tests that were conducted at baseline and at week 12, all other study assessments as well as safety assessments were carried out at baseline, visit 3 (week 4), visit 4 (week 8), and visit 5 (week 12). Before any study-related procedures, all subjects gave written informed consent, which specifically indicated that participation was voluntary and could be terminated at any time without giving any reason. The study was approved by the Clinical Research Ethics Committee of the Cork Teaching Hospitals, Cork (ECM 4 (ll) 9 March 2021) and registered at Clinical Trials (NCT04931082) before the first participant was included. The study was conducted in accordance with the ethical principles in the Declaration of Helsinki, the International Council for Harmonization, and E6 R2 Guideline for Good Clinical Practice (ICH GCP). 

### 2.2. Study Population

Healthy males and females aged 21 to 50 years with moderate stress were recruited via advertisements, and using a list of previously reported interested subjects. All subjects lived in the area of Cork, Ireland. Interested subjects were asked to fill in the Cohen’s Perceived Stress Scale (PSS) and the Hospital Anxiety and Depression Scale (HADS) questionnaires for the assessment of their stress levels. A PSS score between ≥14 and ≤26, and a HADS score of ≤ 10 for anxiety and depression were required for enrollment. Subjects within these ranges, who fulfilled all inclusion and exclusion criteria, were asked to participate in the clinical study and were scheduled for their inclusion visit (visit 1). Exclusion criteria included active IBS (according to the Rome IV criteria), any chronic intestinal disease, immunodeficiency disorders or immunosuppressive treatment, any known food allergies or celiac disease, intake of antibiotics within four weeks before the screening visit or any previous psychiatric illness (within five years), consumption of systemic psychotropics, rheumatoid drugs, steroid drugs, regular asthma medications or use of creams containing cortisone within the previous four weeks before the screening visit. Subjects who had been included in any other clinical study with an intake of any investigational product, who were pregnant or had plans to change their current diet and/or exercise regime during the participation of the trial, had any malignant disease or other end-organ diseases that were judged to interfere with the study, or those with a history of heavy caffeine consumption (>400 mg/day) were also excluded. Any intake of antibiotics, and/or intake of other probiotic supplements or other food supplements which could interfere with the study was also prohibited. Subjects were informed that deviating from the study procedures would lead to premature withdrawal. 

### 2.3. Study Product

The subjects were randomly assigned to one of two treatment arms: LPHEAL9 or placebo (1:1). The randomization list was computer-generated with a block size of four by an external statistician. The probiotic treatment arm received one capsule daily consisting of *Lactiplantibacillus plantarum* HEAL9 (LPHEAL9, HEAL9™, DSM 15312) at a dose of 10^10^ CFU (10B CFU) per day, maize starch, maltodextrin (bulking agents), and magnesium stearate (processing and anti-caking aid). The placebo arm received one capsule per day with an identical appearance containing only maize starch and magnesium stearate. Both capsules were white vegetable capsules (hydroxypropyl methylcellulose and titanium dioxide). The study product was packed externally, and specific personnel not involved in the study were responsible for the labeling. Study products were handed out at baseline, week 4, and week 8, with a four-week supply on each occasion. The study was double-blind, and the subjects and personnel involved in the study did not know which product (LPHEAL9 or placebo) was distributed. The subjects were asked to return any unused study product at the next visit, which was used to assess compliance. The subjects were asked to refrain from using supplements and foods containing probiotics, supplements with dietary fiber or known to affect cognition (e.g., Vitamin B12, fish oil, etc.) during their participation in the study. 

### 2.4. Assessment of Stress, Mood, Cognition, and Sleep

Cohen’s Perceived Stress Scale (PSS) was used to assess the subjective feelings of stress throughout the study [11]. The PSS is composed of 10 questions on feelings and thoughts during the past month and measures the degree to which situations in one’s life are assessed as stressful. The individual scores can range from 0 to 40 with higher scores indicating a higher perceived stress. For cut-off values, levels below 13 are considered as low stress, 14–26 as moderate stress, and 27–40 as high perceived stress [12]. Cohen’s PSS was evaluated at all five visits of the study. 

For the assessment of mood states, the short form of the Profile of Mood State (POMS) questionnaire was used [13]. It is a psychological rating scale that assesses transient and distinct mood states, based on seven different subscales (Tension–Anxiety, Depression–Dejection, Anger–Hostility, Vigor–Activity, Fatigue–Inertia, and Confusion–Bewilderment, and Friendliness). A higher total score indicates a worse outcome for mood disturbance, whereas a lower score indicates little or no mood disturbance. Both the total score and the different subscales were evaluated at visits 2 through 5 during the study. 

The Pittsburg Sleep Quality Index (PSQI) questionnaire assesses sleep quality over a one month period [14]. It is composed of 10 questions, the answers to which provide both a composed score and five different sub-scores (duration of sleep, sleep disturbance, sleep latency, daytime dysfunction, and overall sleep efficiency). Subjects were considered as having poor sleep quality if the total score was >5 and good sleep quality with a value of ≤ 5, thus lower scores indicate a better sleep quality.

The Competency Assessment (COMPASS) battery of different cognitive assessment tools was used to investigate cognitive performance before and after the intervention. COMPASS was developed by the Brain, Performance, and Nutrition Research Centre, Northumbria University, and is commercially available (https://www.cognitivetesting.co.uk, accessed on 2 August 2023). It has been used extensively to assess the cognitive benefits of dietary supplements, nutritional compounds, and probiotics. Five tests from COMPASS were used in the study. Attention and vigilance were tested using the four choice reaction time test (FCRT) and rapid visual information processing test (RVIP). In both, an increased percentage of correct responses and decreased reaction time indicate an improvement. The FCRT shows arrows on a screen pointing at different locations, and the subjects are requested to indicate the direction of the arrows as fast as possible. The RVIP shows a series of numbers one by one in quick succession and the subjects are requested to indicate whenever they see three odd or even numbers displayed in a row. Working and spatial memory were tested using the numeric working memory test (NWM) and the Computerized Corsi block test. In the NWM test, subjects are shown one number at a time and instructed to memorize the numbers. Once the series is complete the procedure is repeated, including other numbers, subjects indicate whether the number displayed was in the previous list or not. Improvements are measured by increased accuracy and decreased reaction time. The Corsi block test shows subjects nine blue squares on a black background. The squares change to red, black, and blue again in different sequences, and the subjects recall the response by using the cursor to click the squares. This is repeated in up to fifteen levels as long as the participant selects enough correct responses. An improvement is measured as an increase in span score (average of the last 3 correct responses in a row). Short-term memory was assessed with a word recall test. Subjects are shown one word per second and once 15 words are displayed, they are given 60 s to write down as many words as they remember (immediate recall). Improvement is measured as an increase in accuracy. The Paired-Associate Learning test (PAL) from the Cambridge Neuropsychological Test Automated Battery (CANTAB^®^; Cambridge Cognition, https://cambridgecognition.com, accessed on 2 August 2023) was also included in the study. PAL assesses learning and memory [15]. White boxes are presented on the screen and open in a randomized order. Some of the boxes display a certain pattern, and subjects are requested to learn and remember a specific pattern and where it is located. The number of patterns increases with each level. The test terminates when the subject does not identify the correct patterns within 10 trials. A decrease in errors indicates improvement.

### 2.5. Awakening Cortisol and Analysis of Biomarkers in Blood 

The Cortisol Awakening Response (CAR) is the change in cortisol concentration that occurs in the first hour after waking from a night’s sleep [16]. It is different from the rest of the cortisol output during the day and has been related to stress and other psychological symptoms [17]. Cortisol levels were measured in saliva samples collected by passive drooling at T0, T30, T45, and T60 min after waking on the days of the visits at the clinic. The subjects were not allowed to brush or floss their teeth and eat or drink anything until after all samples had been collected. The samples were brought to the clinic and thereafter stored at −80 °C until analysis. Analysis of cortisol was performed with ELISA (High sensitivity salivary cortisol, Salimetrics, State College, PA, USA), according to the manufacturer’s instructions. 

Blood samples for assessing biomarkers were collected at baseline and after 12 weeks. Serum and plasma were separated by centrifugation for 10 min at 3000 rpm at 4 °C. The samples were thereafter stored at −80 °C until analysis. 

The biomarkers assessed were Transforming Growth Factor β 1 (TGF-β1), galectin-3, fractalkine/CX3CL/CX3CL1, brain-derived neurotrophic factor (BDNF), tryptophan, L-kynurenine, and high-sensitivity C-reactive protein (hs-CRP). 

ELLA Protein Simple plex (BioTechne, Dublin, Ireland) was used to analyze BDNF (#898072, detection limit 9.4–36,000 pg/mL), fractalkine (#898143, detection limit 56.8–86,710 pg/mL), TGF-β1 (#898477, detection limit 20.8–12,684 pg/mL), and galectin-3 (#898088, detection limit 3.11–4741 pg/mL) in serum. 

Plasma levels of tryptophan and L-kynurenine were measured with commercially available ELISA kits (ImmuSmol, Bordeaux, France). The L-kynurenine kit (BA E-2200) had a detection limit of 45.7 ng/mL, and the tryptophan kit (BA E-2700) had a measuring range of 0.73–250 µg/mL. Hs-CRP was measured by the medical service at Skåne University Hospital (University and regional laboratories in Region Skåne, Sweden) using an immune turbidimetric analysis with a detection range between 0.16 and 10 mg/L. 

### 2.6. Safety

Blood samples for safety parameters (clinical biochemistry and hematology) were collected at visit 1 (screening), visit 2 (baseline), and visit 5 (12 weeks). The samples were analyzed by Eurofins Biomnis, Dublin, Ireland. Safety was also assessed via monitoring of adverse events (Aes) and physical examinations. The subjects were also questioned about their medication history during the screening visit and thereafter queried at each visit about the use of any concomitant medications. 

### 2.7. Statistical Methods

As this was a novel exploratory study, there was no pre-existing data to complete an a priori sample size calculation. 

The results presented in this paper are for the intention to treat (ITT) population if not otherwise indicated. ITT includes all randomized subjects with at least one reported consumption of any of the products. The per protocol population (PP) includes all randomized subjects with complete data for the primary outcomes, and a minimum of 80% reported compliance in terms of the amount of product consumed with no major protocol deviations. All subjects, who took at least one dose of the study product, were included in the safety population. 

All analyses were two-sided at a 5% significance level. However, since it is an exploratory study, some *p*-values of >0.05 were also reported. For each endpoint, if there was no statistically significant difference(s) at baseline between the two groups, a mixed two-way between–within groups ANOVA was used to determine whether there was a statistically significant change over time in the endpoint between the two groups from baseline (visit 2) to the end of the intervention (visit 5). For some variables, outliers were identified (data that were greater than 1.5 box lengths from the edge of the boxplot) and removed from the analysis. Sensitivity analyses were furthermore conducted using two-way between–within groups ANOVA to determine whether there was a statistically significant change over time in the endpoint between the two groups from baseline (visit 2) to week 4 (visit 3) to week 8 (visit 4) until the end of the intervention (week 12; visit 5). The difference between the groups in terms of change from baseline to each timepoint (week 4, week 8, and week 12) separately was also evaluated using an unpaired *t*-test. The non-parametric Mann-Whitney U Test was implemented as an alternative to parametric estimates when the assumptions of those methods were in doubt, or when parametric inference was impossible. An analysis of the change from baseline within a group was conducted using paired *t*-test or the non-parametric test Wilcoxon Signed-Rank test or Friedman’s test. 

If the data were categorized, McNemar’s test was used to determine if there was a statistically significant within-group change in terms of the proportion of subjects in each category from baseline to the end of the intervention, and chi-square analysis was used to evaluate differences between the groups at each time point. All analyses were conducted using the SPSS IBM V 28.0.

## 3. Results

### 3.1. Study Population

**Figure 1:**
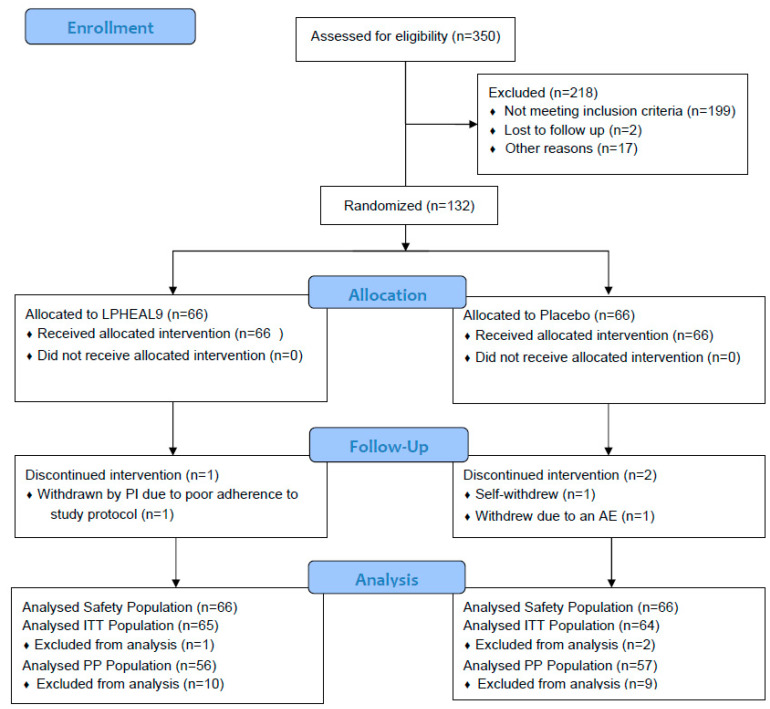
Participant flow chart. Abbreviations: ITT, intention to treat; PP, per protocol; PI, primary investigator.

Three hundred and fifty subjects were screened for eligibility; out of them, 199 did not meet the inclusion criteria and 19 subjects were not able to be included due to other reasons (Figure 1). Thus, 132 subjects were randomized into the study; 66 were allocated to the LPHEAL9 group and 66 were allocated to the placebo intervention group. In the LPHEAL9 group, 65 of the subjects finished the study, while 64 finished in the placebo group (ITT population). The PP population consisted of 113 subjects: 56 subjects in the LPHEAL9 group and 57 in the placebo. The reasons for not being included in the LPHEAL9 PP population were one or several of the following: missed visit window (*n* = 4), intake of <80% of the study product (*n* = 2), did not return study product (*n* = 1), AE (*n* = 2), and intake of prohibited medication (*n* = 2). In the placebo group, subjects were excluded from the PP population due to one or several of the following reasons: missed visit window (*n* = 2), intake of <80% of the study product (*n* = 1), did not return study product (*n* = 2), AE (*n* = 2), pregnancy (*n* = 1), and intake of prohibited medication (*n* = 2).

The ratio between female/male were equal in both LPHEAL9 and placebo groups, 65% vs. 67%. The mean (min–max) age was 35.2 (21–52) years, and the BMI was 26.4 (19–47) kg/m^2^ (Table 1).

### 3.2. Perceived Stress and Awakening Cortisol

**Figure 2:**
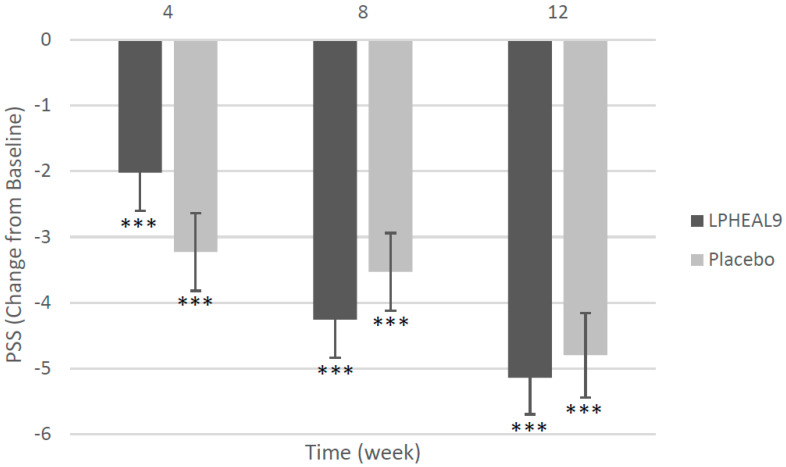
Perceived stress as measured by Cohen’s Perceived Stress Scale (PSS). Both LPHEAL9 and placebo groups experienced a significant decline in PPS from baseline to week 12. Within group difference compared to baseline *** *p* < 0.001 (Wilcoxon Signed-Rank Test).

Perceived stress reduced significantly in both groups during the intervention period of 12 weeks with no significant difference between the groups (Figure 2, Table S1). The mean reduction in PSS score after 12 weeks of intake was 28.3% in the LPHEAL9 group and 26.6% in the placebo group (*p* = 0.69 between groups). All subjects had moderate stress at baseline (PSS mean 17.9; min 14; max 26), while 50.8% in the LPHEAL9 group and 48.4% in the placebo group had low stress after 12 weeks of intervention (a PSS below 14; NS difference between groups). 

Stress was also evaluated by measuring the cortisol awakening response. The salivary cortisol levels were measured four times during one hour after the subjects woke up, at baseline, and after 4, 8, and 12 weeks of intervention. The area under the curve (AUC) was determined together with the maximum level (Cmax) for each time point. Both groups displayed significantly lower AUC and Cmax over the intervention period, with the largest reduction observed at week 12 for the LPHEAL9 group and at week 8 for the placebo group with no significant differences between the groups (Table S1). Normally the cortisol levels increase in the morning, but for about 38% of the subjects, the cortisol level did not increase over 2.5 nmol/L at the baseline measurement (non-responders). Performing the analysis including only the responders did not change the results. Only looking at the first sample taken after the subject woke up (T0-sample) showed that the salivary cortisol level was significantly reduced at week 12 compared with baseline in the LPHEAL9 group (*p* = 0.039) but not in the placebo group (*p* = 0.525).

### 3.3. Mood and Quality of Sleep

Mood was evaluated by the Profile of Mood States (POMS) questionnaire. The mean total mood disturbance score was 20.5 at baseline for the LPHEAL9 group and 18.4 for the placebo group (NS difference) (Table S2). For both groups, the mean total mood score reduced significantly over time, i.e., showed improved mood, and at week 12, the groups had mean scores of 9.1 and 10.6, respectively (*p* = 0.161 between groups). All seven subscales improved significantly in the LPHEAL9 group after 12 weeks, while in the placebo group, three of the subscales did not change over the course of the study (Confusion–Bewilderment, Depression–Dejection, and Friendliness). For one of the sub scales, Confusion–Bewilderment, there was a trend for benefitting from LPHEAL9 consumption (mean change from baseline to week 12: −1.63) compared to placebo consumption (−0.47, *p* = 0.051). In addition, for two of the mood subscales, Anger–Hostility and Depression–Dejection, there were trends for interaction between time and group, with an overall favor of LPHEAL9 vs. placebo (mixed ANOVA, *p* = 0.086 and *p* = 0.088, respectively). For Anger–Hostility, the score reduced equally in both groups at week 4, but at weeks 8 and 12, a larger decrease in the LPHEAL9 group was observed (*p* = 0.072 between groups at week 8). The scores for Depression–Dejection behaved in a similar way with larger reductions seen at week 8 (*p* = 0.079 between groups) and at week 12 for the LPHEAL9 group. 

The quality of sleep was evaluated using the questionnaire Pittsburg Sleep Quality Index (PSQI). The PSQI total score reduced significantly over time in both groups and no difference between the groups was observed (Table S3). The PSQI total score can be divided into good sleep (score ≤ 5) or poor sleep (score > 5). At baseline, 55% of the subjects in the LPHEAL9 group and 61% in the placebo group had poor sleep (NS difference between groups). The percentage of subjects having poor sleep decreased over time, with a tendency of improved sleep in the LPHEAL9 group vs. placebo group. At week 8, the corresponding figures were 28% and 44%, respectively (*p* = 0.069 between groups), and at week 12, 29% and 44%, respectively (*p* = 0.087 between groups) (Chi-square analysis). The subscales of the PSQI showed a significantly improved overall sleep quality only within the LPHEAL9 group (*p* = 0.037), while no difference was seen in the placebo group. There was also a significant improvement in daytime dysfunction within the LPHEAL9 group (*p* < 0.001), and a trend for an improvement in the placebo group (*p* = 0.052). 

### 3.4. Cognition

Six cognition tests were conducted at baseline and after 12 weeks of intervention. For two of these tests, all subjects scored high already at baseline and only minor changes over time were observed (Four Choice Reaction time and Computerised Corsi block; results not presented). For the other four tests, differences between the groups were measured (Table S4). 

**Figure 3:**
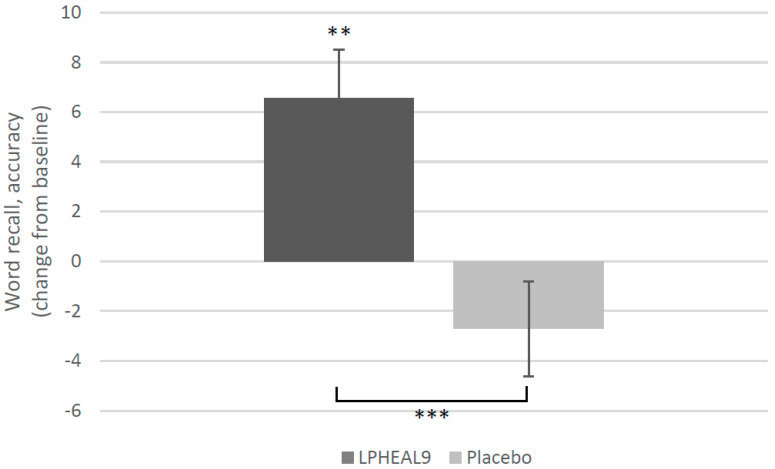
Word recall test, mean change (SEM) from baseline to week 12 in accuracy (%). Within-group differences compared to baseline ** *p* < 0.01 (Wilcoxon Signed-Rank Test) and between-group differences *** *p* < 0.001 (mixed two-way between–within groups ANOVA).

A significant effect of LPHEAL9 on short-term memory was observed in the word recall test (Figure 3). The mean % accuracy increased significantly in the LPHEAL9 group from 42.8 at baseline to 49.3% after 12 weeks (*p* = 0.003, ITT population). In contrast, the mean % accuracy decreased in the placebo group from 46.35 to 43.65% and a significant difference between the groups was observed (*p* < 0.001) in both the ITT and PP population.

Working and spatial memory were tested using the numeric working memory test (NMW). Intake of LPHEAL9 reduced the reaction time from baseline to week 12 compared to intake of placebo (*p* = 0.081 in ITT and *p* = 0.049 in PP, Mann–Whitney U test). A total of 70% of the subjects had an accuracy above 90% at baseline, and after 12 weeks all subjects in both groups had an accuracy above 90% (NS difference). 

Attention and vigilance were studied using the rapid visual information processing test (RVIP), where the subjects had to indicate when three odd or even numbers appeared in a row from the numbers appearing in quick succession on the screen. Also, in this test, intake of LPHEAL9 was seen to cause a greater reduction in reaction time compared to placebo (*p* = 0.051 in ITT and 0.039 in PP). There was no difference in accuracy between the groups, but it increased significantly from about 33% at baseline to 41% and 43% in the LPHEAL9 group and placebo group, respectively, at week 12. 

**Figure 4:**
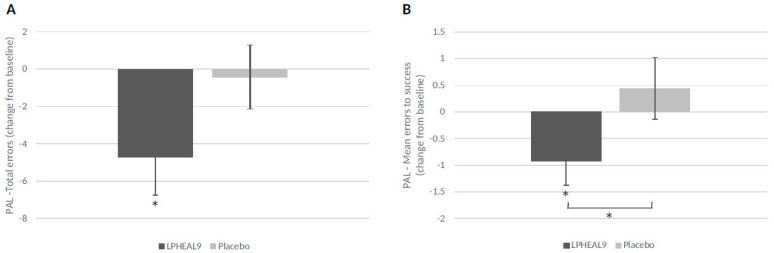
Paired associate learning (PAL): Mean (SEM) change from baseline to week 12 in total errors (**A**) and mean errors to success (**B**). Within-group differences compared to baseline * *p* < 0.05 (Wilcoxon Signed-Rank Test) and between-group differences * *p* < 0.05 (mixed two-way between–within groups ANOVA, outliers removed).

In the paired associate learning test (PAL), the total and mean errors to success were evaluated. Following 12 weeks of LPHEAL9 treatment, the total errors decreased significantly compared to baseline for both the ITT (*p* = 0.031) and PP populations (*p* = 0.028), while the total errors did not change significantly over time in the placebo group (Figure 4A). However, the between-group differences were not significant (*p* = 0.113 in ITT and *p* = 0.068 in PP). The mean errors to success decreased significantly, with −0.92 for the LPHEAL9 group (*p* = 0.031) compared to a non-significant increase of 0.44 for the placebo group (Figure 4B). The difference between the LPHEAL9 and placebo groups was significant for both the ITT (*p* = 0.048) and PP population (*p* = 0.045). 

### 3.5. Blood Biomarkers

Blood biomarkers were analyzed at baseline and after 12 weeks of intervention. The tryptophan metabolism was evaluated by measuring the tryptophan and kynurenine levels. No significant changes were observed for the LPHEAL9 group, while both tryptophan and kynurenine levels increased significantly from baseline to week 12 in the placebo group (Table 2). For tryptophan, the increase over time was significantly higher in the placebo group compared to the LPHEAL9 group. Different inflammation markers were also measured. The CRP level was below the detection limit (0.3 mg/L) for 10% of the subjects. Excluding these subjects, as well as subjects with increased CRP levels (>10 mg/L), indicating an ongoing infection/other non-healthy state, showed that the CRP level did not change over time. Non-significant changes over time were also observed for brain-derived neurotrophic factor and galectin-3 for both groups (Table S5). The pro-inflammatory marker fractalkine increased in the placebo group (*p* = 0.09), while a small decrease was observed in the LPHEAL9 group (54.3 pg/mL vs. −0.78 pg/mL; *p* = 0.13 between groups). The anti-inflammatory marker TGF-β decreased significantly in the placebo group (*p* = 0.03), but the level was unchanged in the LPHEAL9 group (*p* = 0.176 between groups). 

### 3.6. Intake of Study Product and Safety

Compliance was overall good in the study and in the ITT population; the mean intake of the LPHEAL9 product was 98.5%, while the mean intake was 99.0% for the placebo product during the 12 week intervention period. 

Thirty-nine adverse events were reported during the study, 20 in the LPHEAL9 group and 19 in the placebo group. Two adverse events were possibly related to the intake of LPHEAL9, and they were mild in intensity (a non-pathological increase in alanine aminotransferase levels). Thus, intake of LPHEAL9 was found to be safe and well tolerated.

## 4. Discussion

The present exploratory study was conducted to gain insight into the effect of LPHEAL9 on the gut–brain axis. Measurements of stress, cognition, mood, and sleep were investigated after intake of LPHEAL9 or placebo for 12 weeks. The primary findings in this study indicated a positive impact on cognitive performance, especially memory functions, after intake of LPHEAL9 compared to placebo. These results may be connected to the improvements over time seen by LPHEAL9 in sleep quality, overall mood, and awakening cortisol. 

Perceived stress (PSS) was reduced significantly in both the LPHEAL9 and the placebo groups, with no difference between the groups. Thus, a large placebo effect on subjects with moderate stress was seen. This observation aligns with a study testing *L plantarum* DR7 in a similar population [9]. However, the administration of *L. paracasei* Lpc-37 to subjects with chronic stress was found to significantly decrease PSS in the probiotic group compared to the placebo group in another study [18]. Some earlier studies also indicate a significant decrease in stress levels after intake of probiotics in subjects with low stress. Messaouidi et al. [19] observed a significant difference in PSS in a subgroup with the lowest urinary free cortisol levels at baseline after intake of *L. helveticus* R0052 and *B. longum* R0175 in comparison to placebo. In an earlier study on LPHEAL9, lower levels of cortisol were seen after the Trier Social Stress Test (TSST) in the LPHEAL9 group compared to the placebo group. These results were even more pronounced in a subgroup that did not have chronic stress on the day of performing the TSST [4]. In the current study, a significant reduction in the awakening cortisol level over time was observed in both groups, while the cortisol level directly after awakening (T0) significantly decreased only in the LPHEAL9 group at week 12. 

The connection between stress and sleep has been known for a long time. One of the causes of this connection is the “fight or flight” response arising from stressful situations. This leads to increased heart rate and blood pressure, as well as increased levels of cortisol and adrenaline, responses that greatly affect sleep quality and duration of sleep [20]. Several studies have investigated the impact of sleep on different lactobacilli strains. A randomized and placebo-controlled study in healthy but stressed students found that intake of *L. plantarum* JYLP-326 was able to relieve symptoms of anxiety, depression, and insomnia after three weeks of consumption [21]. Similarly, *L. casei* Shirota was found to improve sleep quality in terms of sleepiness when rising and sleep length in healthy but stressed adults [22]. However, a probiotic mixture given to 38 healthy volunteers for six weeks did not show a significant difference in sleep between the probiotic and placebo groups measured using PSQI [23]. Like the present study, they did find improvements within the probiotic group in both sleep and mood. In the present study, both groups were found to have significantly improved PSQI, while two of the sub-scores significantly improved only within the LPHEAL9 group. The significantly improved results in overall sleep quality, as well as in daytime dysfunction, may be connected to the significantly decreased immediate awakening cortisol (T0) level found only in the LPHEAL9 group at week 12 compared to baseline. The overall improved sleep in both groups could be connected to the significantly decreased levels of perceived stress, which were similar for both groups over the course of the study. 

Mood is defined as a transient state of mind, and one of the most common methods used to measure it is the POMS questionnaire. Apart from the overall score obtained from this test, there are six pre-defined sub-scores (Anger–Hostility, Confusion–Bewilderment, Depression–Dejection, Fatigue–Inertia, Tension–Anxiety, Vigor–Activity, and Friendliness). In the current study, all the sub-scores were significantly improved within the LPHEAL9 group from baseline to week 12. The placebo group reported minor but still significant changes within four of the six sub-scores. For three of the sub-scores, there was a trend for a difference in favor of LPHEAL9 compared to placebo, namely Anger–Hostility, Depression–Dejection, and Confusion–Bewilderment. Similar results were obtained in a study with a probiotic blend given to healthy individuals, where significant within-group differences were observed only for the above-mentioned sub-scores related to the “negative” subscales of mood [23]. Another study investigating the impact of a dual-strain probiotic on healthy subjects with a non-pathological mood disturbance showed a close to significant improvement in the overall POMS level, as well as within-group improvements in the negative aspects of mood only for the probiotic group [24]. Decreased levels of the negative subscales of POMS are interpreted as an improved mood. 

In the present study, the subjects were challenged using cognition tests before and after treatment. Generally, many cognition tests are used to evaluate if the subject has cognitive impairment, but in a study with healthy and relatively young subjects, as in the present case, it may not be surprising that some cognition tests were too easy (four choice reaction time and Corsi blocks). But the more difficult tests (numeric working memory, rapid visual information processing, paired associate learning, and word recall) were found to be applicable to evaluate differences due to treatment. One of the most significant results in the present study is that intake of LPHEAL9 improved working memory compared to the placebo group in the word recall test. The effect of LPHEAL9 on working memory agrees with a similar study that tested the effect of *L. plantarum* DR7 on subjects with moderate stress for 12 weeks [9], and a study investigating the intake of a probiotic mixture showing an increased stress-induced working memory in the probiotic group but not in the placebo group [10]. A positive effect on memory after consumption of LPHEAL9 was also seen in the numeric working memory test, where there was a significantly larger reduction in the reaction time for the LPHEAL9 group compared to the placebo group. Learning and visuospatial memory were studied in the paired associate learning test, where LPHEAL9 treatment caused a significant reduction in the mean errors to success compared to the placebo group. A trend for a difference in total errors was also observed in the PP population (*p* = 0.068, between groups) in favor of LPHEAL9, which agrees with an earlier study that observed a larger reduction in PAL total errors after intake of *Bifidobacterium longum* 1714 compared to placebo [8]. 

Different mechanisms can explain the effects of LPHEAL9 on cognition. It is known that LPHEAL9 survives the passage through the gastrointestinal tract [25,26,27] and has the ability to adhere to human mucosa cells that is dependent on a mannose-binding mechanism [28,29]. Thus, LPHEAL9 has the possibility to affect factors that influence cognition.

The cognitive function can be influenced by components derived from the metabolism of tryptophan such as kynurenine, as shown in a study with *L. plantarum* 299v, where intake of this probiotic strain decreased kynurenine levels and improved cognitive function in subjects with depression [6]. However, in the present study on subjects with moderate stress, no changes in the tryptophan and kynurenine levels were found in the LPHEAL9 group, while both parameters increased significantly in the placebo group. The increase in tryptophan level was significantly higher in the placebo group compared to the LPHEAL9 group. In agreement with these results, an earlier 8-week study with *L. casei* Shirota YIT 9029 or placebo administered to fifty-one healthy medical students under academic examination stress showed a significant increase in tryptophan from baseline to one day before the exam in the placebo group compared to the probiotic group (*p* < 0.05). Likewise, the kynurenine value also increased at the same time (*p* = 0.07), while no significant changes were observed in the probiotic group [30]. It is difficult to explain the results of increased levels in the placebo group, while no differences were detected in the probiotic group, but one factor that may lower the tryptophan and kynurenine levels in plasma could be that gut bacteria metabolize tryptophan into indole and indolic compounds [31]. Indoles have several health benefits and can enhance barrier function, regulate intestinal immune tolerance, and act as neuroprotective compounds [31]. The potential ability of LPHEAL9 to metabolize tryptophan to indoles is currently unknown, but other lactobacilli strains have been shown to carry this capability [31,32]. In the paper by Kato-Kataoka et al. [30], it was suggested that the lower tryptophan levels in the probiotic group could have been caused by enhanced serotonin biosynthesis. 

In the present study, the biomarker brain-derived neurotrophic factor (BDNF) was measured. BDNF is important for the survivability and growth of neurons and thus affects learning and memory [33]. It has been shown that intake of two *Bifidobacterium* strains for 12 weeks by older adults (≥65 years) increased the BDNF level significantly compared to placebo [34]. However, no change over time in BDNF after intake of LPHEAL9 was found in the moderately stressed subjects, aged 21 to 52 years, included in the present study. Previous studies including relatively young subjects support the present results of no differences in BDNF [35,36,37]. 

Elevated stress has been connected to increased pro-inflammatory properties; therefore, three pro-inflammatory markers were used in this study. Galectin-3 has been shown to be involved in different diseases associated with chronic inflammation, cancer, and type 2 diabetes [38]. To our knowledge, the effect of probiotics on galectin-3 in moderately stressed subjects has not been studied previously. No clear results for this biomarker were found in the present study, probably due to the subjects included being healthy with normal levels of galectin-3 at baseline. The same theory can be applied to CRP, where no changes were observed over time. Fractalkine is another pro-inflammatory marker that is prominent in the intestinal epithelium [39]. It is produced in response to different inflammatory stimuli and has been shown to be involved in conditions such as atherosclerosis, cancer, rheumatoid arthritis, and IBD [40]. In an earlier study on LPHEAL9, the level of fractalkine was significantly lower in the LPHEAL9 group compared to the placebo group after an acute stress test (TSST) [4]. In the present study, there was a trend for a similar result, with a higher fractalkine level found in the placebo group (*p* = 0.085), while the fractalkine level remained unchanged in the LPHEAL9 group (*p* = 0.883) after 12 weeks of intervention compared to the baseline (*p* = 0.133 between groups). In the present study, the results may have been influenced by the stressful situation of facing a cognition test, since the blood sample for fractalkine analysis was taken just before the initiation of these tests. The anti-inflammatory marker TGF-β was also measured, and comparable to the results of the pro-inflammatory marker fractalkine, a change in TGF-β was seen in the placebo group (*p* = 0.027), while no change over time was observed in the LPHEAL9 group. The more stable inflammatory status in the probiotic group compared to the placebo group may be one underlying mechanism of the observed positive effect of LPHEAL9 on cognition. 

Different gastrointestinal metabolites, such as short-chain fatty acids, branched-chain amino acids (BCAA), and peptidoglycans, can also affect the gut–brain axis [41]. It is not known how LPHEAL9 influences the different metabolites. However, a recent publication showed that intake of LPHEAL9 together with *L. paracasei* 8700:2 for six months by children with celiac disease autoimmunity affected the fecal metabolome, significantly decreasing the BCAA threonine level and increasing the 4-hydroxyphenylacetate level compared to the placebo group [42]. 

This study has some limitations. As this was an exploratory trial without any pre-existing data as a basis for power calculations, the study was underpowered for some variables, which restricted the interpretation of the data. Furthermore, a fecal microbiota analysis could have added data for a suggestive mechanism of the actions behind the observed effects of LPHEAL9. A final limitation of this study was that placebo effects were observed for several variables. However, this may be difficult to overcome when evaluating subjective measurements in healthy and fairly young individuals and when the baseline scores for some of the endpoints were near normal, making it difficult to demonstrate an improvement. In future studies, measurements less influenced by a placebo response should preferably be selected, for example, by including additional performance challenges.

## 5. Conclusions

The present study was designed to evaluate the effect of the probiotic bacteria *Lactiplantibacillus plantarum* HEAL9 on moderately stressed but otherwise healthy individuals. Several different endpoints were included in the study, and the results showed that intake of LPHEAL9 for 12 weeks had a positive and significant impact on cognition. More specifically, the study showed a significant improvement in learning and working memory after the consumption of LPHEAL9 compared to placebo, potentially by ameliorating aspects of mood and improving sleep. These effects may be linked to the stabilization of inflammatory biomarkers and to a significantly lower awakening level of cortisol in the probiotic group compared to the baseline. 

## Figures and Tables

**Table 1:** Summary of demographic data at inclusion (mean, range).

	ITT LPHEAL9 (*n* = 65)	ITT Placebo (*n* = 64)	PP LPHEAL9 (*n* = 56)	PP Placebo (*n* = 57)
Females	65%	67%	66%	67%
Age, year	35.5 (21–52)	34.9 (21–50)	36.0 (21–52)	34.6 (21–50)
BMI, kg/m^2^	26.3 (19–40)	26.6 (19–47)	26.6 (20–40)	26.8 (20–47)

No significant differences at inclusion between intervention groups.

**Table 2:** Kynurenine and tryptophan levels in plasma at baseline, week 12, and change over time in the ITT population (median, range). LPHEAL9 (*n* = 64); placebo (*n* = 62).

	Baseline	12 Weeks	Change	*p*-Value ^#^
Kynurenine (ng/mL)				
LPHEAL9	439.8 (210.3–1276)	450.8 (187.7–1295)	−3.9 (−603.3–413.4)	
Placebo	438.1 (176.7–1695)	497.5 (213.7–1173)	32.0 (−521.2–397.8) *	0.234
Tryptophan (μg/mL)				
LPHEAL9	9.80 (4.81–16.08)	10.11 (4.84–16.35)	−0.67 (−8.90–9.36)	
Placebo	9.90 (4.84–19.53)	11.53 (4.75–20.39)	2.31 (−11.92–12.06) ***	0.003

# Between-group differences: independent samples from Mann–Whitney U Test. * *p* < 0.05; *** *p* < 0.001. Within-group: Wilcoxon Signed-Rank Test.

## Data Availability

The data presented in this study are available upon request from the corresponding author.

## Supplementary Materials

The following supporting information can be downloaded at: https://www.mdpi.com/article/10.3390/nu15153466/s1, Table S1: Perceived stress scale (PSS) and awakening cortisol (AUC and Cmax, mmol/L) at baseline, week 4, 8, and 12 in the ITT population; Table S2: Profile of mood scores (POMS) at baseline, week 12 and change over time in the ITT population; Table S3: Quality of sleep (PSQI) at baseline, week 12 and change over time in the ITT population; Table S4: Cognition tests at baseline, week 12, and change over time in the ITT population; Table S5: Inflammation markers in serum at baseline, week 12, and change over time in the ITT population.
