# Supplementary material for: Intake of Lactiplantibacillus plantarum HEAL9 Improves Cognition in Moderately Stressed Subjects: A Randomized Controlled Study
Source: Nutrients. 2023 Aug 5;15(15):3466. doi: 10.3390/nu15153466 (PMC10421450; doi:10.3390/nu15153466)
Supplement: Supplementary file 1 [file nutrients-15-03466-s001.zip › nutrients-2532178-supplementary.pdf]

Table S1. Perceived stress scale (PSS) and awakening cortisol (AUC and Cmax, mmol/L) at baseline, week 4, 8 and 12 in the ITT population (mean (SEM), LPHEAL9 (n=63-65), Placebo (n=62-64))

	**Baseline**	**4 weeks**	**8 weeks**	**12 weeks**
PSS Total score[1]				
LPHEAL9	18.05 (0.37)	16.03 (0.66)***	13.78 (0.52)***	12.91 (0.58)***
Placebo	17.98 (0.42)	14.75 (0.70)***	14.45 (0.64)***	13.19 (0.68)***
Awakening cortisol AUC[2]				
LPHEAL9	835.3 (48.9)	711.9 (38.6)**	756.8 (47.6)*	687.2 (43.8)**
Placebo	936.5 (48.8)	869.8 (62.4)	773.0 (49.8)***	806.8 (55.4)*
Awakening cortisol Cmax[2]				
LPHEAL9	17.78 (1.12)	15.05 (0.82)*	16.35 (0.99)	14.97 (0.90)*
Placebo	19.96 (1.04)	18.67 (1.54)*	17.04 (1.06)**	17.71 (1.18)*

[1] No significant differences between groups at any timepoint in change from baseline. Within group differences compared to baseline were significantly different for all timepoints for both groups ***p<0.001 (Paired t-test).

[2] No significant differences between groups at any timepoint in change from baseline. Within group differences compared to baseline * p<0.05, ** p<0.01, ***p<0.001 (Related samples Wilcoxon signed rank test).

Table S2. Profile of mood scores (POMS) at baseline, week 12 and change over time in the ITT population (mean (SEM), LPHEAL9 (n=65), Placebo (n=64))

	**Baseline**	**12 weeks**	**Change baseline-w12**	**P, comparison between groups[1]**
Total mood disturbance				
LPHEAL9	20.50 (2.02)	9.10 (2.05)	-11.40 (2.26)***	
Placebo	18.38 (2.11)	10.59 (2.43)	-7.79 (2.23)***	0.161
Anger-Hostility				
LPHEAL9	5.52 (0.43)	3.38 (0.42)	-2.13 (0.51)***	
Placebo	5.31 (0.56)	3.75 (0.48)	-1.56 (0.44)***	0.211[2]
Confusion-Bewilderment				
LPHEAL9	5.73 (0.47)	4.10 (0.43)	-1.63 (0.44)***	
Placebo	5.23 (0.44)	4.77 (0.47)	-0.47 (0.51)	0.051
Depression-Dejection				
LPHEAL9	3.23 (0.40)	2.33 (0.35)	-0.90 (0.42)*	
Placebo	2.89 (0.41)	2.40 (0.42)	-0.49 (0.44)	0.221[3]
Fatigue-Inertia				
LPHEAL9	9.40 (0.57)	7.37 (0.55)	-2.04 (0.61)*	
Placebo	9.43 (0.59)	7.19 (0.64)	-2.25 (0.63)***	0.759
Tension-Anxiety				
LPHEAL9	8.04 (0.56)	4.96 (0.44)	-3.08 (0.56)***	
Placebo	7.73 (0.52)	5.76 (0.53)	-1.97 (0.55)***	0.269
Vigour-Activity				
LPHEAL9	11.42 (0.48)	13.04 (0.64)	1.62 (0.60)*	
Placebo	12.23 (0.51)	13.28 (0.60)	1.05 (0.51)*	0.472
Friendliness				
LPHEAL9	15.57 (0.47)	17.12 (0.50)	1.54 (0.48)**	
Placebo	15.79 (0.53)	16.68 (0.54)	0.90 (0.52)	0.345

[1] Between group differences: Independent-Samples Mann-Whitney U Test

[2] p=0.086 (Between groups Mixed two-way between-within group ANOVA including all timepoints)

[3] p=0.088 (Between groups Mixed two-way between-within group ANOVA including all timepoints)

* $p<0.05$, ** $p<0.01$, ***$p<0.001$ (t-test, within group difference)

Table S3. Quality of sleep (PSQI) at baseline, week 12 and change over time in the ITT population (mean (SEM), LPHEAL9 (n=65), Placebo (n=64))

	Baseline	12 weeks	Change baseline-w12	P, comparison between groups
Total score				
LPHEAL9	6.05 (0.33)	4.94 (0.32)	-1.11 (0.33)***	
Placebo	6.58 (0.36)	5.36 (0.33)	-1.22 (0.29)**	0.958
Duration of sleep				
LPHEAL9	0.85 (0.10)	0.82 (0.10)	-0.03 (0.11)	
Placebo	1.08 (0.10)	0.89 (0.10)	-0.19 (0.09)	0.307
Sleep disturbance				
LPHEAL9	1.25 (0.05)	1.06 (0.06)	-0.18 (0.07)*	
Placebo	1.20 (0.06)	1.09 (0.05)	-0.11 (0.05)**	0.288
Sleep latency				
LPHEAL9	1.35 (0.11)	1.05 (0.09)	-0.31 (0.10)**	
Placebo	1.59 (0.12)	1.14 (0.12)	-0.45 (0.12)***	0.343
Daytime dysfunction				
LPHEAL9	1.00 (0.07)	0.72 (0.07)	-0.28 (0.09)***	
Placebo	0.88 (0.06)	0.67 (0.07)	-0.20 (0.07)	0.727
Sleep efficiency				
LPHEAL9	0.54 (0.09)	0.43 (0.09)	-0.11 (0.10)	
Placebo	0.70 (0.12)	0.59 (0.11)	-0.11 (0.10)	0.994
Overall sleep quality				
LPHEAL9	1.05 (0.06)	0.86 (0.09)	-0.18 (0.09)*	
Placebo	1.08 (0.06)	0.95 (0.08)	-0.12 (0.09)	0.591

Between group differences: Independent-Samples Mann-Whitney U Test

* p<0.05, ** p<0.01, ***p<0.001 (Within group, Friedman’s test, all timepoints included)

Table S4. Cognition tests at baseline, week 12 and change over time in the ITT population (mean (SEM)).

	Baseline	12 weeks	Change baseline-w12	P, comparison between groups
Word recall, % accuracy				
LPHEAL9 (n=65)	42.8 (1.9)	49.3 (2.2)	6.6 (1.9)**	
Placebo (n=64)	46.3 (1.8)	43.6 (2.0)	-2.7 (1.9)	<0.001
Numeric working memory test				
Accuracy (%)				
LPHEAL9 (n=65)	92.3 (1.0)	91.0 (2.2)	-1.2 (2.4)	
Placebo (n=64)	89.1 (2.4)	92.2 (1.2)	3 (2.3)	0.374[1]
Reaction time (msec)				
LPHEAL9 (n=65)	1271.9 (36.6)	1145.4 (29.1)	-126.2 (24.9)***	
Placebo (n=64)	1241.3 (41.0)	1152.1 (40.4)	-79.7 (24.1)**	0.081[1]
Rapid visual information processing test				
Accuracy (%)				
LPHEAL9 (n=65)	33.81 (2.10)	41.38 (2.41)	7.58 (1.98)***	
Placebo (n=64)	33.16 (2.72)	43.40 (2.77)	10.23 (2.12)***	0.361
Reaction time (msec)				
LPHEAL9 (n=65)	535.7 (9.7)	508.8 (10.3)	-26.9 (14.7)	
Placebo (n=64)	520.1 (9.2)	504.6 (15.7)	-15.5 (16.7)	0.051[2]
Paired associate learning				
Total Errors				
LPHEAL9 (n=63)	17.6 (2.6)	12.9 (2.1)	-4.7 (2.0)*	
Placebo (n=60)	15.0 (2.6)	14.7 (2.5)	-0.4 (1.7)	0.113
Mean errors to success				
LPHEAL9 (n=63)	3.6 (0.6)	2.7 (0.5)	-0.9 (0.5)*	
Placebo (n=60)	2.8 (0.7)	3.3 (0.6)	0.4 (0.6)	0.048[2]

Between group differences: Mixed two-way between-within group ANOVA

[1] Mann-Whitney U Test

[2] Outliers removed

* p<0.05, ** p<0.01, ***p<0.001 (Within group, Wilcoxon signed rank test).

Table S5. Inflammation markers in serum at baseline, week 12 and change over time in the ITT population (mean (SEM)).

	**Baseline**	**12 weeks**	**Change baseline-w12**	**P, comparison between groups**
BDNF (ng/ml)				
LPHEAL9 (n=65)	33.64 (1.18)	32.87 (1.03)	-0.77 (0.85)	
Placebo (n=63)	33.59 (0.85)	32.63 (0.95)	-0.96 (0.60)	0.857
TGF-β (ng/ml)				
LPHEAL9 (n=64)	59.33 (1.86)	59.24 (1.84)	-0.09 (1.75)	
Placebo (n=63)	58.76 (1.63)	55.63 (1.58)	-3.13 (1.38)*	0.176
Fractalkine (pg/ml)				
LPHEAL9 (n=65)	1150 (23.77)	1149 (24.17)	-0.78 (17.18)	
Placebo (n=61)	1193 (30.33)	1247 (40.21)	54.33 (29.05)	0.133
Galectin-3 (ng/ml)				
LPHEAL9 (n=65)	8.85 (0.24)	8.71 (0.24)	-0.14 (0.18)	
Placebo (n=63)	8.84 (0.26)	8.81 (0.28)	-0.03 (0.21)	0.690

Between group differences: Independent-Samples t-test

* p<0.05 (Within group, paired t-test)
